# Association between inflammatory bowel disease and chronic obstructive pulmonary disease: a systematic review and meta-analysis

**DOI:** 10.1186/s12890-019-0963-y

**Published:** 2019-10-28

**Authors:** Gonzalo Labarca, Lauren Drake, Gloria Horta, Michael A. Jantz, Hiren J. Mehta, Sebastian Fernandez-Bussy, Erik Folch, Adnan Majid, Michael Picco

**Affiliations:** 1 Facultad de Medicina, Universidad San Sebastian, Lientur 1457, 4100000 Concepcion, Chile; 2 A.T. Still University Kirksville College of Osteopathic Medicine, Kirksville, MO USA; 3 Division of Gastroenterology, Hospital Regional Grant Benavente, Concepcion, Chile; 4 Division of Pulmonary and Critical Care Medicine, University of Florida-Gainesville, Gainesville, USA; 5 Division of Pulmonary and Critical Care Medicine Mayo Clinic Jacksonville, Florida, USA; 6 Division of Pulmonary and Critical Care, Massachusetts General Hospital, Boston, USA; 7 Division of Thoracic surgery and Interventional Pulmonology, Beth Israel Deaconess Medical Center, Boston, USA; 8 Division of Gastroenterology, Mayo Clinic Jacksonville, Florida, USA

**Keywords:** Lung diseases, Obstructive, Pulmonary disease, Chronic obstructive, Inflammatory bowel diseases

## Abstract

**Introduction:**

There is evidence of an association between inflammatory bowel disease (IBD) and lung conditions such as chronic obstructive pulmonary disease (COPD). This systematic review and meta-analysis explored the risk of new onset IBD in patients with COPD and new onset COPD in IBD patients.

**Methods:**

We performed a systematic review of observational studies exploring the risk of both associations. Two independent reviewers explored the EMBASE, MEDLINE, LILACS and DOAJ databases, and the risk of bias was evaluated using the ROBBINS-I tool. Data from included studies was pooled in a random effect meta-analysis following a DerSimonian-Laird method. The quality of the evidence was ranked using GRADE criteria.

**Results:**

Four studies including a pooled population of 1355 new cases were included. We found association between new onset IBD in COPD population. The risk of bias was low in most of them. Only one study reported tobacco exposure as a potential confounding factor. The pooled risk ratio (RR) for a new diagnosis of IBD in COPD patients was 2.02 (CI, 1.56 to 2.63), *I*^*2*^ *=* 72% (GRADE: low). The subgroup analyses for Crohn’s disease and ulcerative colitis yielded RRs of 2.29 (CI, 1.51 to 3.48; *I*^*2*^ *=* 62%), and 1.79 (CI, 1.39 to 2.29; *I*^*2*^ *=* 19%.), respectively.

**Discussion:**

According to our findings, the risk of new onset IBD was higher in populations with COPD compared to the general population without this condition. Based on our analysis, we suggest a potential association between IBD and COPD; however, further research exploring the potential effect of confounding variables, especially cigarette smoking, is still needed.

**Review register:**

(PROSPERO: CRD42018096624)

## Introduction

Inflammatory bowel disease (IBD), which commonly includes both Crohn’s disease (CD) and ulcerative colitis (UC), is related to several gastrointestinal manifestations, including an increased risk of colorectal cancer. Multisystemic involvement is reported in 25% of IBD patients [1]. However, the clinical association between IBD and chronic respiratory conditions, such as chronic obstructive pulmonary disease [2], is currently vague [3, 4].

The intestine and airway are related in that both are derived from the same embryological structure and share similarities in their in columnar epithelium, mucous glands, goblet cells and lymphoid tissue in their submucosal layers [5]. Moreover, the loss of immunological tolerance and the immunological disruption that results in response to environmental triggers are common pathways described in both intestinal and airway disease mechanisms [4]. There is evidence that patients with UC and CD show patterns of subclinical alveolitis on bronchoalveolar lavage and transbronchial sample biopsy 60% of the time, [6, 7] although the clinical significance of this phenomenon is still unknown.

IBD may develop from a genetic predisposition of the intestinal mucosa and/or environmental “triggers”, leading to immunologic dysregulation between pro-inflammatory and anti-inflammatory cytokines that causes a shift towards a pro-inflammatory state [8, 9]. The gut microbiome may also have a role in this process; in recent years, several publications have reported a potential contribution from the gut microbiome and its role in the immune response [10, 11].

IBD is associated with COPD. In a meta-analysis of population-based studies, Crohn’s disease was associated with an increased risk of death by COPD (standardized mortality ratios 2.55, CI, 1.19–5.47) [12]. A potential mechanism for this association is lung-gut cross-talk [10]. Inflammation may lead to the production of autoimmune antibodies against elastin or in response to antigens produced during oxidative stress. Such oxidative stress may be produced by cigarette smoking [13]. IL-6 and TGF-β may drive cross-organ Th-17-induced inflammation [14]. Additionally, IL-13 may drive aberrant natural killer T-cells and macrophage responses across organs [15].

Additionally, tobacco exposure is an important risk factor associated with CD and is likely protective in UC, as the age at diagnosis in UC is older in former smokers [16]. Smoking is also an important risk factor associated with respiratory disease. Therefore, it is possible that smoking may be a direct link between the development of IBD in chronic lung disease or may act as a significant confounding variable of any reported association of lung disease with IBD [17]. Cigarette exposure is also related to changes in pulmonary function and intestinal disruption, especially in the colon where cigarette smoke causes colon shortening and increased levels of angiogenesis and pro-inflammatory cytokines, including TNF- α, IFN-y and TGF- β [10, 11].

Respiratory manifestations of IBD are infrequently reported in the literature, and case reports lack a biological explanation [18]. The aim of this systematic review and meta-analysis is to explore the risk of new onset IBD in patients with COPD.

## Methods

This systematic review and meta-analysis was performed according to the Preferred Reporting Items for Systematic Reviews and Meta-Analyses [19, 20] statement and the Meta-Analysis Of Observational Studies in Epidemiology [21, 22] protocol that was previously published in the PROSPERO database (review number: CRD42018096624).

### Literature search

One of the review authors (GL) performed the literature search without language restriction in January 2019, and an update was performed in September 2019. We identified studies from the following sources: MEDLINE, EMBASE, Lilacs, Epistemonikos, Directory of Open Access Journals (DOAJ) and Google Scholar. We also hand-searched the proceedings from major respiratory conferences of the European Respiratory Society (ERS; 2014 to 2018), American Thoracic Society (ATS; 2014 to 2018), American College of Chest Physicians (2014 to 2018), American Gastroenterological Association (AGA: 2014 to 2018), European Crohn’s and Colitis Organization (ECCO, 2014 to 2018), and the Crohn’s & Colitis Congress (2014 to 2018). Details of these strategies are reported in the Additional file 1: S1 Appendix.

Finally, we searched the reference lists of all primary studies.

### Inclusion criteria

We included adult participants with either COPD or IBD at baseline. COPD participants were included upon meeting the GOLD criteria with a FEV1/FVC < 70% [23]. We also searched for participants with a history of COPD in each database using ICD-9 or similar criteria. For IBD, we included participants with a diagnosis according to clinical and pathological criteria or a data record of IBD (ulcerative colitis or Crohn’s disease) according to the ICD-9 criteria. To explore the potential bi-directional influence of either COPD on IBD or IBD on COPD, we included studies that explored both associations. As the exposure, we evaluated the time of environmental exposure among the participants and defined the time of no exposure or standard risk of either COPD or IBD as a comparison. As the outcome, we defined the relative risk of new onset IBD (Crohn’s colitis or ulcerative colitis) or COPD. We restricted our inclusion to observational studies (either retrospective or prospective cohort, cross-sectional and case-control studies) who reported the prevalence or incidence of new cases of either IBD or COPD. Finally, we excluded studies of patients with COPD associated with other diseases or another airway disease (asthma, bronchiolitis, pulmonary hypertension, and others).

### Data collection and analysis

#### Selection of studies

Two review authors (GL and LD) evaluated titles and abstracts of the search results and independently included and excluded studies based on the inclusion criteria. A third review author (SFB) was involved in resolving any discrepancies. A table defining the characteristics of the excluded studies was then created based on the manuscript selection.

Two review authors (GL and LD) independently extracted outcome data from the eligible studies, and one review author (GL) transferred the data into Review Manager version 5.3.

### Assessment of the risk of bias in the included studies

Two review authors (GL and LD) assessed the risk of bias independently for each study using the criteria outlined in the ROBINS-I tool for nonrandomized studies [24]. Any disagreement was resolved through discussion. The risk of bias was assessed according to the following domains: (1) bias due to confounding variables such as smoking (smoking history or lack of information on smoking from the databases), sex (male), average age and observation bias (patients routinely seeing a doctor for lung disease were more likely to be diagnosed with IBD compared to controls not frequently followed for medical care); (2) bias due to selection of the participants (database registry, inpatient database or prospective cohort); (3) bias due to classification of the intervention (report of the exposure to environmental factors, especially tobacco exposure, in each included study); (4) bias due to deviation from the intended interventions (deviation from the pre-defined cohort to explore IBD or an environmental exposure); (5) bias due to missing data; (6) bias in the measurement of the outcome and (7) bias in the selection of reported results.

### Data management

The qualitative synthesis includes data from observational studies that were not eligible for meta-analysis or randomized control trials.

We used participants as the unit of analysis and defined the proportion of new cases of either IBD or COPD as the number of events reported with a new diagnosis of the complementary condition (IBD, COPD or vice versa). We extracted data on either the prevalence or incidence of new cases reported in each study. For those studies that report associations with other diseases, we manually extracted only data that referred to either IBD or COPD. As a comparator, we extracted the number of new cases of IBD or COPD in the control population reported in each study.

Data from new cases of IBD or COPD in both the case and control groups were pooled using the DerSimonian-Laird method for meta-analyses. Dichotomous data and pooled data were then expressed as risk ratios (RRs) and 95% confidence intervals (CI) for each analysis that was included. Every meta-analysis followed the randomized analysis mode using Review Manager version 5.3 software.

Heterogeneity was evaluated using the *I*^*2*^ statistic to measure heterogeneity among the studies in each analysis. We defined an *I*^*2*^ > 50% as substantial heterogeneity. In the case of substantial heterogeneity, we explored possible causes by pre-specified subgroup analyses according to 1) Crohn’s disease or ulcerative colitis, 2) study type (population-based cohort/ non cohort studies) and 3) tobacco exposure (reported/not reported).

For tobacco exposure, we extracted data concerning current, former and non-smokers. A current smoker was defined as a participant who self-reported tobacco use. A former smoker was defined as a participant who declared a positive tobacco history and stopped smoking at least 6 months prior to the study. A non-smoker was defined as a participant who self-reported no tobacco use. All conditions were defined at diagnosis of IBD.

Publication bias was evaluated through a visual inspection of a funnel plot. Finally, we reported the main results using a summary of findings table and rated the quality of evidence according to GRADE criteria [25]. (www.gradepro.org).

## Results

**Fig. 1:**
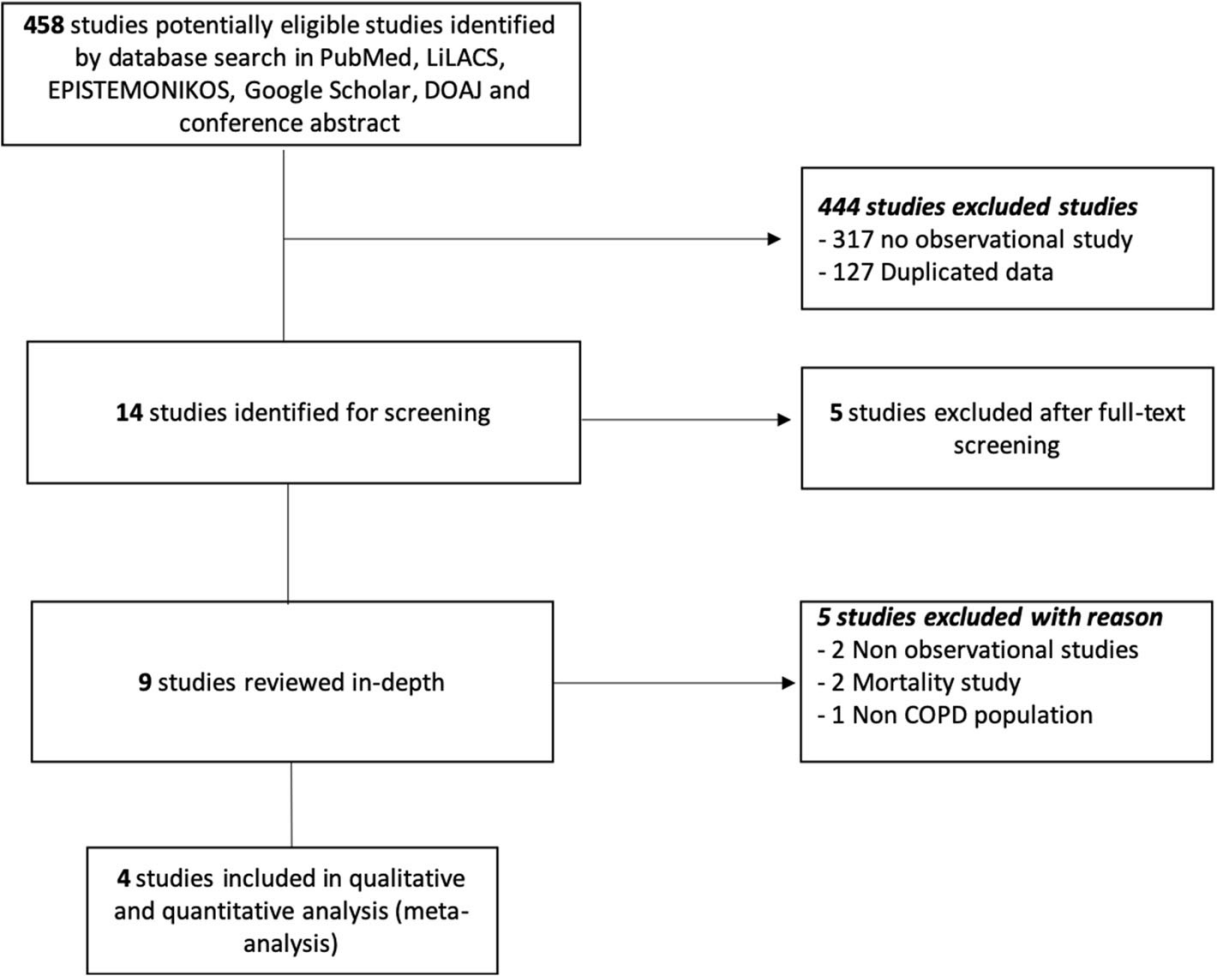
PRISMA Flow

We identified 458 studies from different databases and society meetings; 9 of them were selected after screening. Five studies were excluded with reason [26–30] and four were included for the qualitative and quantitative analysis; three were published papers [18, 31, 32] and one was a meeting abstract [33]. Figure 1 describes the summary of the literature search according to PRISMA standards. Data regarding articles excluded with reason are reported in the Additional file 1: Table S1.

### Qualitative analysis

Four studies included a pooled participant size of 325,731 patients with a total of 1355 new cases. The average age of the participants was 61 years old. Two studies reported age as either a range or a percentage [18, 32]. All studies were retrospective. Three were population-based studies [18, 32, 33], and one was a case control study. Raj et al. included a population of 2192 participants in a respiratory clinic, and only 588 of them were included in the COPD population [31]. Brassard and Kang reported the incidence of new cases of IBD, and both Ekbom and Raj reported the prevalence of new cases of IBD. Regarding location, one study was from Sweden, one was from Canada, one was from South Korea and one was from the U.K. All studies included a population with a diagnosis of COPD at baseline, and the average follow-up ranged between 2.0 and 3.9 years. A summary of the characteristics of the studies is shown in Table 1.

**Table 1:** Characteristic of included studies. IBD: Inflammatory Bowell Disease, CD: Crohn Disease, UC: ulcerative colitis, COPD: Chronic obstructive pulmonary disease, U.K: United Kingdom; N.R: non-reported

Author	Year	Country	Cohort Size	Study design (prospective/retrospective)	Mean IBD, (Age)	Male, (%)	Tobacco	Included diseases
Raj A.	2008	U. K	2.192	Retrospective	CD: 60; UC: 61	CD: 46; UC: 41	CD: 23% current and 46% former. UC: 5% current and 50% former	Asthma; COPD; Chronic cough; Bronchiectasis; Chronic bronchitis
Brassard P.	2014	Canada	280.082	Retrospective	Ages broken up by range	CD: 69; UC: 72	N. R	Asthma; COPD
Ekbom A.	2008	Sweden	180.239	Retrospective	Ages broken up by range	N. R	N. R	COPD
Kang E.	2018	South Korea	20.042	Retrospective	N. R	N. R	N. R	COPD

### Quality assessment

We reported a low risk of bias in one study. The main causes of a high risk of bias were confounding variables, the selection of the participants (unclear diagnosis or ICD-9 criteria without a confirmation test) and selection of reported results. Three studies reported bias due to confounding variables such as smoking [18, 32, 33], one reported bias due to the selection of the participants [31], and two reported bias in the selection of the reported results [31, 33]. A full quality analysis for the involved studies using the ROBINS-I criteria is shown in Table 2.

**Table 2:** Risk of bias from included studies using ROBINS-1 criteria

Study	Bias of confounding variables	bias in selection of participant into study	Bias in classification from intended intervention	Bias due to deviation from intended intervention	Bias due to missing data	Bias in measurement of outcomes	Bias in selection of reporting results
Raj A.	Low	High	Low	Low	High	Low	High
Brassard P.	High	Low	Low	Low	Low	Low	Low
Ekbom A.	High	Low	Low	Low	High	Low	Low
Kang E.	High	Low	Low	Low	High	High	High

### Tobacco exposure

Tobacco exposure was reported in one study. Raj and colleagues [31] explored the prevalence of IBD in patients with airway diseases. The prevalence of non-smokers was 45% in patients with UC and 31% in patients with CD; 50% of patients with UC and 46% of patients with CD were reported to be former smokers. Finally, the prevalence of current smokers was 5% in UC patients and 23% in CD patients. The authors compared the expected prevalence of UC and CD to the prevalence in the general population.

### Outcome report

#### Risk of IBD in COPD populations

The risk of IBD was increased in all studies. Raj et al. reported an increased odds ratio of 5.26 for CD and an odds ratio of 3.57 for UC, Ekbom et al. reported a 1.83-fold higher risk of UC and a 2.72-fold higher risk for CD, Brassard et al. reported an increased incidence of 55% for CD and COPD patients, and the incidence of ulcerative colitis was 30% higher in the COPD population. Kang et al. also reported an increased incidence of 61% for CD and 31% for UC.

**Fig. 2:**
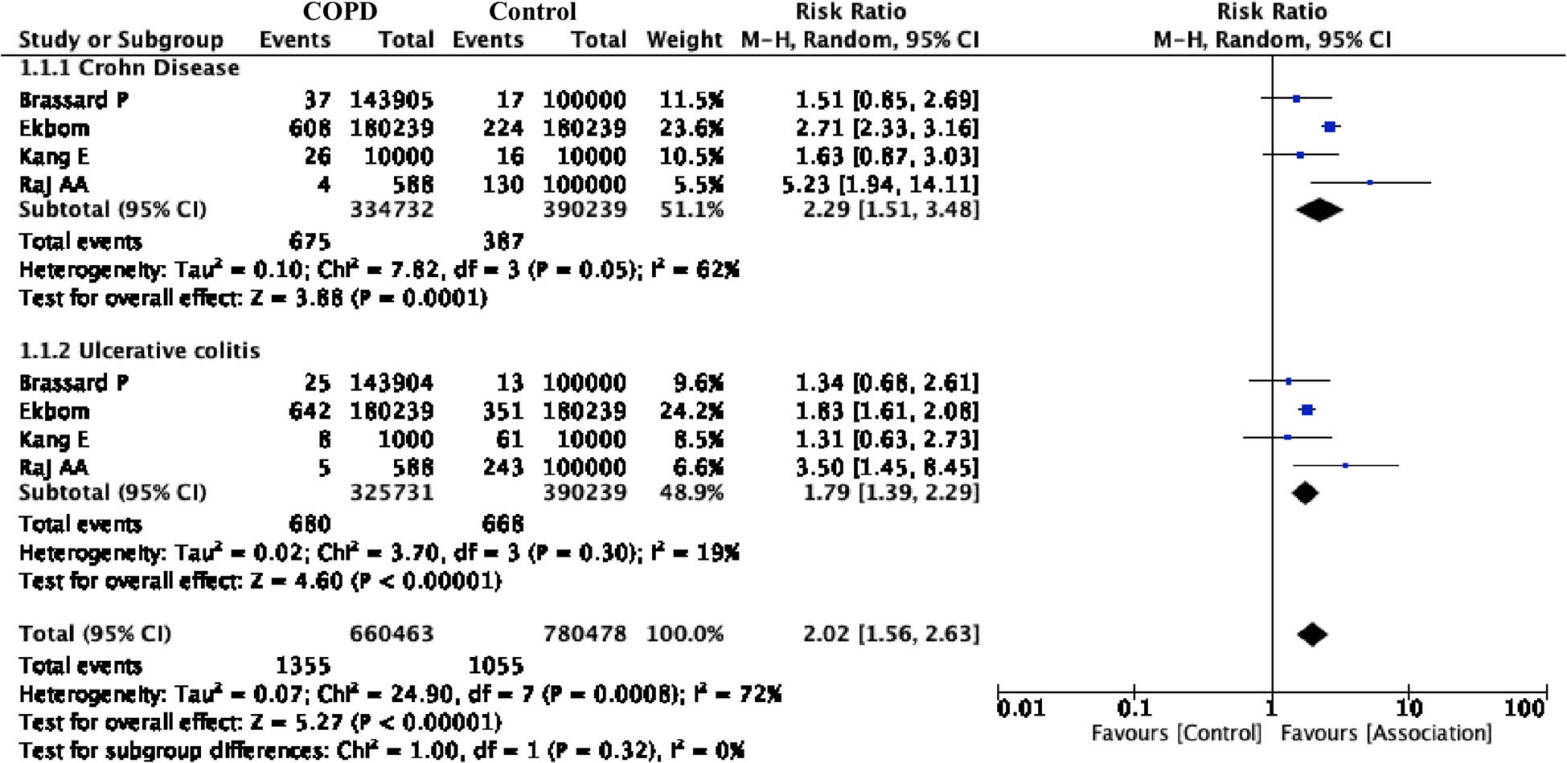
Comparison 1. Risk of IBD in COPD patients and subgroup analysis according to Crohn’s disease (CD) or Ulcerative colitis (UC)

The pooled data from the four studies, including a total of 1355 new cases of IBD and 1055 cases in the control group, were included in our meta-analysis. The association between COPD and IBD was consistently increased throughout the included studies. The pooled RRs for a new diagnosis of IBD in the COPD population were 2.02 (CI, 1.56 to 2.63), *I*^*2*^ = 72% (*p =* 0.0008) (Fig. 2). There was serious publication bias according to the funnel plots (Additional file 1: Figure S1). We rated this evidence as low using the GRADE approach due to the risk of bias, inconsistencies and a plausible cofounding effect. A summary of findings (SoF) table and an explanation of the GRADE approach are presented in Table 3.

**Table 3:** Summary of findings table using the GRADE approach

Association between IBD and COPD						
Patient or population: COPD Setting: Intervention: IBD Comparison: Control						
Outcomes	Anticipated absolute effects^*^ (95% CI)		Relative effect (95% CI)	№ of participants (studies)	Certainty of the evidence (GRADE)	Comments
	Risk with Control	Risk with Airways disease				
Risk of IBD in COPD patients (IBD-COPD)	128 per 100,000	355 per 100,000 (263 to 481)	RR 2.02 (1.56 to 2.63)	660,463 (4 observational studies)	⨁⨁⨁◯ Low ^a,b,c^	Despite association between IBD and COPD was consistently elevated. Lack of confounding analysis by tobacco exposure would suggest a spurious effect
*The risk in the intervention group (and its 95% confidence interval) is based on the assumed risk in the comparison group and the relative effect of the intervention (and its 95% CI). CI: Confidence interval; RR: Risk ratio						
GRADE Working Group grades of evidence High certainty: We are very confident that the true effect lies close to that of the estimate of the effect Moderate certainty: We are moderately confident in the effect estimate: The true effect is likely to be close to the estimate of the effect, but there is a possibility that it is substantially different Low certainty: Our confidence in the effect estimate is limited: The true effect may be substantially different from the estimate of the effect Very low certainty: We have very little confidence in the effect estimate: The true effect is likely to be substantially different from the estimate of effect						

RR: Relative ratio, CI: Confidence interval. IBD: Inflammatory Bowel Disease, COPD: Chronic Obstructive Pulmonary Disease. CD: Crohn’s Disease; UC: Ulcerative colitis

Explanations

a. Risk of bias due to confounding variables, selection of participants (unclear diagnosis or ICD-9 criteria without a confirmation test) and selection of reported results

b. Type of IBD (CD or UC) was the main explanation for heterogeneity. However, residual heterogeneity was low for CD and high for UC

c. Serious publication bias after funnel plot inspection

#### Heterogeneity analysis

The association between COPD and either CD or UC was consistent across studies. The pooled data specifically for Crohn’s disease included 675 new cases and yielded an RR of 2.29 (CI, 1.51 to 3.48), *I*^*2*^ *=* 62%, (*p =* 0.05) (Fig. 2). In this analysis, the residual heterogeneity was considered high. We also explore potential explanations for the residual heterogeneity according to study type, and found an RR of 12.04 (CI, 1.32–3.15; *I*^*2*^ = 66%) for population-based cohort studies compared to 5.23 (CI, 1.94–14.11; I2 = not applicable) for non-cohort studies. (Additional file 1: Figure S2).

The pooled data for ulcerative colitis included 680 new cases and yielded an OR of 1.79 (CI, 1.39–2.29), *I*^*2*^ = 19% (*p =* 0.30). In this analysis, residual heterogeneity was explained by study type, showing an RR of 1.79 (CI, 1.58–2.03; *I*^2^ = 0%) for population-based cohort studies compared to 3.50 (CI, 1.45–8.45; *I*^*2*^ = not applicable) for non-cohort studies (Additional file 1: Figure S3). For tobacco exposure, the first subgroup included the Ekbom and Brassard studies that did not report tobacco history, and the second subgroup included the study conducted by Raj et al. This study was a case-control design and adjusted for tobacco history. A subgroup analysis revealed an RR of 2.31 (CI, 1.48–3.61), *I*^2^ = 52%, (*p* = 0.12) and an intergroup difference of 17%. (Additional file 1: Figure S4).

### Role of the funding source

We found no significant role of the funding source in this systematic review or the included studies.

## Discussion

The main reason for developing this systematic review was to fill in the gap in the literature between observational studies and higher sources of evidence, such as a systematic review. Our findings show that the current evidence regarding the risk of new onset IBD in a population with COPD suggests an association between these conditions.

We found an increased RR in a population with COPD, suggesting a negative impact on colon mucosae that results in increased pro-inflammatory cytokines and angiogenesis factors [10, 13]. Other potential biological mechanisms for this association are related to an increased level of autoimmune antibodies, either from tobacco exposure or from the IBD itself [13, 34, 35]. This chronic injury can contribute to microbiome dysfunction and endothelial barrier dysfunction [36, 37]. Experimental studies also reported a pro-inflammatory status with increased expression of several inflammatory cytokines, such as TNF-a, IL-6, and IL-13, among others [38]. These cytokines increased the levels of lymphocyte Th17, a strong mediator in immune response and neutrophil chemiotaxis [13, 39]. As a result of this inflammatory state, patients with both diseases expressed increased oxidative stress and endothelial dysfunction compared to healthy people [40].

Additionally, cigarette smoke exposure increases the genetic transcription of both VEGF and iNOS, both of which are targets of hypoxia inducible factor (HIF) [3].

Our findings reveal an increased risk of IBD, both CD and UC, in patients with COPD, and the subgroup analysis reported no differences in risk of either disease in population-based cohort studies. Heterogeneity decreased to 0%, which indicates a strong association that is closely related to several reports that describe this association. However, the potential role of cigarette smoking as a trigger for worse prognosis in Crohn’s disease patients is unclear due to a lack of sufficient information. Therefore, based on our analysis, we suggest a potential association between IBD and COPD; however, the quality of the evidence is low, and pulmonologists and gastroenterologists should explore the potential correlation between both conditions and the effect of confounding variables, especially cigarette smoke, age, sex, disease types and inflammatory responses (including microbiota of gut and lung), which may influence the relationship of these diseases. Based on this systematic review, we are able to provide evidence from the epidemiological studies that reported this association, especially in patients with COPD at baseline; however, we found no evidence to support a reciprocal association of an increased risk of COPD in patients with IBD at baseline.

According to previous literature, tobacco history is the main confounder between IBD and chronic respiratory conditions. In a meta-analysis, the association between current smokers and CD had an OR of 1.76 (CI, 1.40–2.22), and former smoking was associated with UC with an OR of 1.79 (CI, 1.37–2.34). However, current smoking had a protective effect on UC compared to controls with an OR of 0.58 (CI, 0.45–0.75) [16]. We carried out an analysis adjusted for studies with tobacco history data and those without tobacco history data. However, the primary studies lacked adjusted analyses, and further exploration between tobacco and the risk of IBD in populations with airway diseases is needed. Only one study reported tobacco status, and we were unable to find an adjusted RR analysis in the primary studies due to the lack of data.

We performed a systematic prognosis review following criteria commonly published by scientific associations, and we also included observational studies to evaluate our clinical question. However, the quality of evidence was graded as low. First, given that UC and CD are such common conditions, the prevalence of both could be potential confounding variables. Second, most studies were retrospective and assigned outcomes according to clinical or epidemiological databases. We found a high or unclear risk of bias in several included studies, with the main reason being bias due to confounding variables. We propose cigarette smoking (current or ex-smoker) as a major cofounding variable in the development of both IBD and COPD; however, smoking as a major confounder appears unjustified based on the available data.

Other variables could include the time from diagnosis (or exposure) to new onset IBD, looking for a “dose–response gradient”, and the patient population, among others. Third, we found moderate heterogeneity between studies, and after a subgroup analysis according to each IBD condition, the residual heterogeneity was still moderate to high, and intergroup heterogeneity was nearly 0%. This could be due to differences between the CD and UC populations, in the age of the included populations or in the risk of bias in the confounding assessment between studies. Unfortunately, data about tobacco history were unreported in most studies, and we were unable to explore this potential confounding source. In our review, we were unable to explore the potential pathophysiological mechanism of this association, and further research incorporating population-based studies in association with translational research can improve the current evidence about this association.

This systematic review is the first to explore the risk of onset of IBD in patients with COPD; we did not find any other systematic reviews exploring this question in the electronic databases or the PROSPERO register of systematic reviews. We evaluated this review following the scientific recommendation for systematic reviews of prognosis; we also included use of the ROBINS-I tool to explore the risk of bias and assessed confidence in the evidence using the GRADE approach for nonrandomized studies [41].

## Conclusion

The risk of developing new onset IBD was higher in the population with COPD populations compared to the general population without this condition. However, the quality of evidence is moderate, and further investigation should be performed to define a potential link between these conditions and to explore the potential confounder impact of cigarette smoking. Further research exploring the potential effect of other confounding variables in prospective cohort studies is needed.

## Supplementary information

**Additional file 1: S1 Appendix**. LITERATURE SEARCH. **Table S1**: Excluded studies with reason. **Figure S1**. Funnel plot Risk of IBD in COPD patients. **Figure S2**. Funnel plot Risk of CD in COPD patients, subgroup analysis by study type. **Figure S3**. Funnel plot Risk of ulcerative colitis in COPD patients, subgroup analysis by study type. **Figure S4**. Subgroup analysis exploring residual heterogeneity in ulcerative colitis. (DOCX 98 kb)

## Data Availability

All data will be available by personal communication with corresponding author.
