# Supplementary material for: Association between inflammatory bowel disease and chronic obstructive pulmonary disease: a systematic review and meta-analysis
Source: BMC Pulm Med. 2019 Oct 28;19:186. doi: 10.1186/s12890-019-0963-y (PMC6819559; doi:10.1186/s12890-019-0963-y)

Supplementary Material.

Supplementary material

S1 Appendix. LITERATURE SEARCH

Source: PubMed (MEDLINE)

Data search: September 11th, 2019

Number of matches: 148

1. “Lung Diseases, Obstructive"[Mesh]
2. “Pulmonary Disease, Chronic Obstructive"[Mesh]
3. “Lung Diseases, Obstructive"[Mesh]

# “Bronchitis, Chronic"[Mesh]

# “Pulmonary Emphysema"[Mesh]

1. “Dyspnea"[Mesh]
2. “COPD” [non-Mesh]
3. “Chronic Obstructive Pulmonary Disease” [non-Mesh]
4. “COAD” [non-Mesh]
5. “Chronic Obstructive Airway Disease” [non-Mesh]
6. “Chronic Obstructive Lung Disease” [non-Mesh]
7. “Airflow Obstruction, Chronic” [non-Mesh]
8. “Airflow Obstructions, Chronic” [non-Mesh]
9. “Chronic Airflow Obstructions” [non-Mesh]
10. “Chronic Airflow Obstruction” [non-Mesh]
11. #1 or #2 or #3 or #4
12. “Gastrointestinal disease” [Mesh]
13. “Inflammatory Bowel Diseases"[Mesh]
14. “Crohn Disease"[Mesh]
15. “Colitis, Ulcerative"[Mesh]
16. #7 or #8 or #9
17. #6 and #10

The MEDLINE strategy will be adapted to the syntax and subject headings of the other

databases.

TRIPDATABASE

Host: Tripdatabase.com

Date of Search: 12 september 2019

#1. (Pulmonary Disease, Chronic Obstructive) (Inflamatory Bowel disease) (risk)

#2. Chronic Obstructive Pulmonary Disease

#3. COPD

Filter: Classification/Clinical trial

LILACS Host: bvsalud.org

Date of Search: 12th September 2019

#1. Pulmonary Disease, Chronic Obstructive

#2. Lung Diseases, Obstructive

#3. COPD

#4. #1 OR #2 OR #3

#5. Inflamatory Bowel Disease

#6. Crohn Disease"[Mesh]

#7. Colitis, Ulcerative

#8. #5 OR #6 OR #7

#9. #4 AND #8

EPISTEMONIKOS

Host: Epistemonikos.org

Date of Search: 07 July 2019

(Chronic Obstructive Pulmonary Disease OR COPD OR Emphysema OR Chronic Bronchitis) AND (Inflammatory bowel disease OR chron disease OR ulcerative colitis)

Filter: Classification/primary studies

Google Scholar

HOST: <https://scholar.google.com/>

Date of Search: 13 September 2019

(Chronic Obstructive Pulmonary Disease OR COPD OR Emphysema OR Chronic Bronchitis) AND (Inflammatory bowel disease OR chron disease OR ulcerative colitis) AND (Risk OR Incidence OR prevalence).

Filter: Classification/primary studies

DOAJ

Host: Doaj. Org

(Chronic Obstructive Pulmonary Disease OR COPD OR Emphysema OR Chronic Bronchitis) AND (Inflammatory bowel disease OR chron disease OR ulcerative colitis)

Filter: Classification/primary studies

S1 Table. Excluded studies with reason

| Author | Year | Reason |
| --- | --- | --- |
| Vutcovici M. | 2016 | Non-control group, outcome measure as mortality |
| Vutcovici M. | 2016 | Non-primary study |
| Bernstein | 2005 | Non-COPD population |
| Cozzi | 2018 | Non clinical outcomes |
| Goyal | 2017 | Non clinical outcomes |

S1 Figure. Funnel plot Risk of IBD in COPD patients


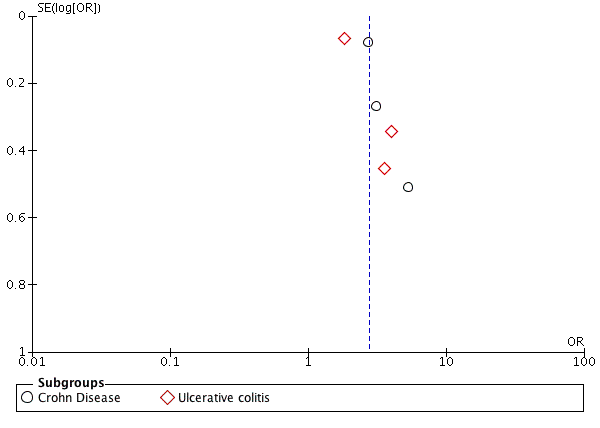


S2 Figure. Funnel plot Risk of CD in COPD patients, subgroup analysis by study type.


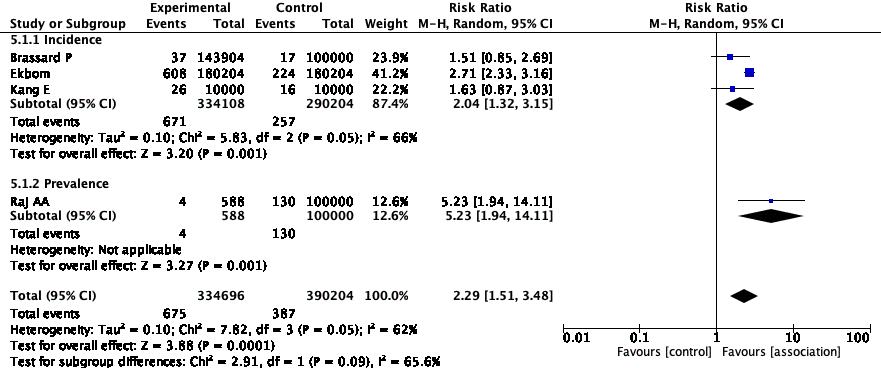


S3. Funnel plot Risk of ulcerative colitis in COPD patients, subgroup analysis by study type.


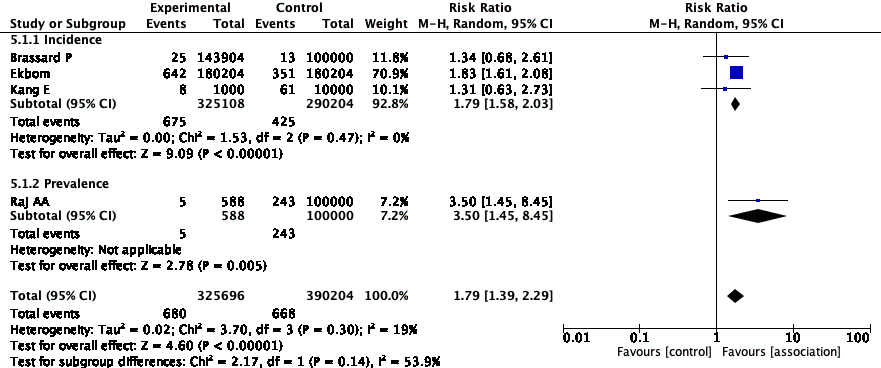


S4. Subgroup analysis exploring residual heterogeneity in ulcerative colitis


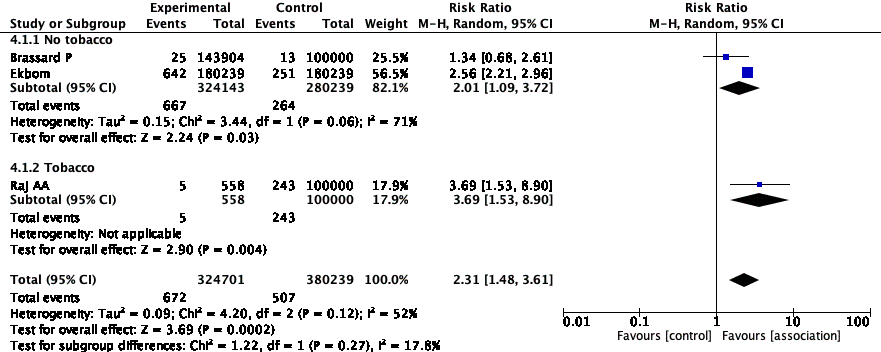

Supplement: Supplementary file 1 — Additional file 1: S1 Appendix. LITERATURE SEARCH. Table S1: Excluded studies with reason. Figure S1. Funnel plot Risk of IBD in COPD patients. Figure S2. Funnel plot Risk of CD in COPD patients, subgroup analysis by study type. Figure S3. Funnel plot Risk of ulcerative colitis in COPD patients, subgroup analysis by study type. Figure S4. Subgroup analysis exploring residual heterogeneity in ulcerative colitis. (DOCX 98 kb) [file 12890_2019_963_MOESM1_ESM.docx]
